# Hydrogel co-loading SO_2_ prodrug and FeGA nanoparticles for enhancing chemodynamic therapy by photothermal-triggered SO_2_ gas therapy

**DOI:** 10.3389/fbioe.2022.1024089

**Published:** 2022-09-29

**Authors:** Qinqin Huang, Meng Lyu, Wenxue Tang, Pengyuan Qi, Hongzhi Hu

**Affiliations:** ^1^ The Research and Application Center of Precision Medicine, The Second Affiliated Hospital, Zhengzhou University, Zhengzhou, China; ^2^ Department of Radiation and Medical Oncology, Hubei Key Laboratory of Tumor Biological Behaviors, Hubei Cancer Clinical Study Center, Zhongnan Hospital of Wuhan University, Wuhan, China; ^3^ Cancer Center, Union Hospital, Tongji Medical College, Huazhong University of Science and Technology, Wuhan, China; ^4^ Department of Orthopaedics, Union Hospital, Tongji Medical College, Huazhong University of Science and Technology, Wuhan, China

**Keywords:** chemodynamic therapy, fega nanoparticles, SO2 gas therapy, glutathione, hydrogel

## Abstract

Chemodynamic therapy (CDT) is an effective anti-tumor method, while CDT alone cannot achieve a good therapeutic effect. Moreover, the overexpression of glutathione (GSH) in tumor cells dramatically limits the efficiency of CDT. Here, we proposed a hydrogel co-loading SO_2_ prodrug and FeGA nanoparticles (NPs) for enhancing CDT by photothermal-triggered SO_2_ gas therapy (FBH) system by mixing benzothiazolyl sulfonates (BTS) and FeGA NPs in a certain ratio and encapsulating them in a heat-sensitive hydrogel. FeGA NPs could accelerate the release of Fe^2+^ under acidic conditions and light, and combine with excess H_2_O_2_ in the tumor for chemokinetic treatment. BTS, as a water-soluble prodrug of SO_2_, can accurately control the release of SO_2_ gas by virtue of the excellent photothermal conversion ability of FeGA NPs and the acidic pH value of tumor site. SO_2_ can not only induce cell apoptosis, but also consume excess GSH in cancer cells and increase the content of reactive oxygen species, which seriously destroyed the redox balance in cancer cells and further promotes the therapeutic effect of Fenton reaction. The intelligent FBH system provided a new approach for the synergistic treatment of CDT and SO_2_ gas, which demonstrated good anticancer effects both *in vivo* and *in vitro*.

## Introduction

Nanoparticles mediated drug delivery and chemodynamic treatment (CDT) is gaining popularity as a form of tumor therapy (Liu et al., 2021; Zhang et al., 2022a; Zhang et al., 2022b; Tang et al., 2022; Zhang et al., 2022). The Fenton or Fenton-like reaction is used by CDT to catalyze the conversion of weakly oxidizing hydrogen peroxide (H_2_O_2_) into strongly oxidizing hydroxyl radical (•OH), which increases intracellular oxidation levels, protein inactivation, DNA necrosis, lipid oxidation, and ultimately induces cancer cell apoptosis (Liu et al., 2018; Lin et al., 2020; Chen et al., 2021; Lin et al., 2021; Xu et al., 2021; Deng et al., 2022). This process takes advantage of the weak acidity and the presence of excess H_2_O_2_ in the tumor microenvironment (Ma et al., 2018; Zhang et al., 2020; Zhu et al., 2022a). CDT has the characteristics of strong specificity and independence, and is suitable for the treatment of tumors deep in the tissue. It is a research hotspot of cancer treatment in recent years. Compared with photodynamic therapy and sonodynamic therapy, CDT does not depend on the oxygen (O_2_) inside the tumor, nor does it require the input of external energy (light energy, ultrasound) (Zhu et al., 2020; Zhu et al., 2021a; Zhu et al., 2022b). It is independent and suitable for the treatment of tumors deep in the tissue, and has a good application prospect in tumor treatment (Li et al., 2017a; Fu et al., 2021). For instance Lin and others showed that copper-based CDT could cause tumor cells to die and release tumor-associated antigens, which could then elicit immunological reactions in the tumor. Nevertheless, since CDT is still in the development stage, the therapeutic efficiency of single CDT therapy is not ideal (Li et al., 2021a; Xin Li et al., 2021; Zhou et al., 2021; Li et al., 2022a; Li et al., 2022b; Guo et al., 2022). On the other hand, glutathione (GSH), as an important antioxidant in cells, can remove the produced •OH, which is the key to cell survival against oxidative stress, this antioxidant defense in cancer cells has become a major obstacle to the efficacy of CDT (Chang et al., 2019; Li et al., 2021b; Hu et al., 2021; Zhou et al., 2021). Depleting the GSH in the tumor microenvironment or using other therapies in combination with CDT is therefore urgently needed to enhance its therapeutic impact. Gas therapy is gaining popularity as a new therapeutic approach for the treatment of tumors (Zhu et al., 2021b). Numerous therapeutic gases, including carbon monoxide, nitric oxide, hydrogen sulfide, oxygen, and sulfur dioxide, have shown promise in the treatment of various diseases, including cancer (Wang et al., 2021; Zhu et al., 2022c). These gases also play important regulatory roles in various physiological processes of cells, tissues, or organisms. Gas therapy is preferable in comparison to chemotherapy, radiation, and other existing treatments due to its enhanced permeability and retention (EPR) impact on tumor tissue (Li et al., 2017b; Zhang et al., 2020; Zhao et al., 2022), which can boost the aggregation of nanoparticles at tumor locations. It also has less drug resistance to tumor cells. Recent research has revealed that sulfur dioxide (SO_2_), long thought to be a hazardous environmental pollutant and a byproduct of industrial processing, also has beneficial benefits on mammals in addition to its poisonous ones. According to reports, SO_2_ has a lot of potential as a gas for the treatment of several diseases, including cancer, inflammation, and cardiac ischemia-reperfusion (I/R) injury (Li et al., 2021a; Li et al., 2021b; Li et al., 2022c). The study also found that SO_2_ can not only cause oxidative damage to various tissues and organs in mice, affecting the activities of various antioxidant enzymes, but also reduce the content of GSH in the tumor microenvironment (Fenton, 1894; Li et al., 2017a; Liang et al., 2021). For example, Wei et al. designed and fabricated a glutathione GSH-reactive SO_2_ polymeric prodrug that synergistically acts with doxorubicin (DOX) on McF-7 ADR in human breast cancer cells. They demonstrated that the released SO_2_ could promote the level of reactive oxygen species (ROS) in tumor cells (Lu et al., 2020). Therefore, combining CDT with SO_2_ gas therapy may enhance the anticancer effect (Shen et al., 2018). However, owing to the rapid diffusion of gas molecules and inadequate tissue penetration, achieving precise SO_2_ release doses in space and time is crucial for on-demand gas therapy. Therefore, nano systems with good biocompatibility and biodegradability for the controllable release of CDT and SO_2_ from tumor tissues are urgently needed.

Having a 1:1 iron/gallate stoichiometry, FeGA is a hexacoordinate complex with a slightly distorted geometry due to the Jahn-Teller effect (Yuan et al., 2021). Each iron center is complexed *via* a total of six iron-oxygen bonds to the benzoic and carboxylate oxygens of four gallate molecules. The five oxygen atoms of gallate act as electron donors due to its pseudo-radical electronic structure, which in turn is beneficial for the reduction of ligands to metals. Gallic acid ligand and iron center exhibit strong metal-ligand exchange coupling, which induces strong electron delocalization throughout the polymer, improving light absorption significantly and turning it dark black while also bringing the spin density of the ionic center closer to the value for high-spin iron (II) (Cun et al., 2022). As a result, the metal-gallate complex’s ionic center serves as its active site, allowing the gallate ligand to transfer electrons to nearby free oxygen molecules, reducing oxygen and producing reactive ROS, and subsequently reducing the iron center (Wang et al., 2020). In acidic environments, the electron loss of Gallate causes degradation of the surrounding ligand field, producing highly oxidized hydroxyl radicals (•OH) that lead to cell death. FeGA nanoparticles (NPs) can also demonstrate a synergistic therapeutic effect of high photothermal therapy (PTT) and CDT, i.e., PTT/CDT, because of its favorable photothermal characteristics [(Li et al., 2019), (Li et al., 2017a)]. GSH, a potent ROS scavenger, can, however, adaptively maintain intracellular redox equilibrium and lessen the cell-killing effects of ROS when it is overexpressed in tumor cells. Developing plausible strategies for GSH depletion in tumors is therefore crucial for promoting the therapeutic effect of the Fenton reaction on tumor death.

Here, we propose a SO_2_ prodrug doped FeGA NPs for enhanced CDT by photothermally triggered gas therapy (FBH) system, which is synthesized by mixing benzothiazolyl sulfonate (BTS) and FeGA NPs in a certain proportion and wrapping in heat-sensitive hydrogel. With the excellent photothermal conversion ability of FeGA NPs and the acidic environment of the tumor site, BTS, as a water-soluble SO_2_ prodrug, can precisely control the release of SO_2_ gas by photothermal. Studies have found that cytotoxic SO_2_ can not only induce apoptosis, but also consume excess glutathione in cancer cells and increase the quantity of reactive oxygen species, thereby severely disrupting the redox balance in cancer cells. FeGA can accelerate the release of Fe^2+^ in an acidic environment and under light, and combine with the excess H_2_O_2_ in the tumor for chemokinetic treatment. At the same time, SO_2_ can deplete GSH in the tumor, which can further assist the therapeutic influence of Fenton reaction on tumor killing. This FBH system provides a new approach for CDT and SO_2_ gas synergistic therapy and shows excellent anticancer effects *in vivo* and *in vitro*.

## Results and discussion

**FIGURE 1:**
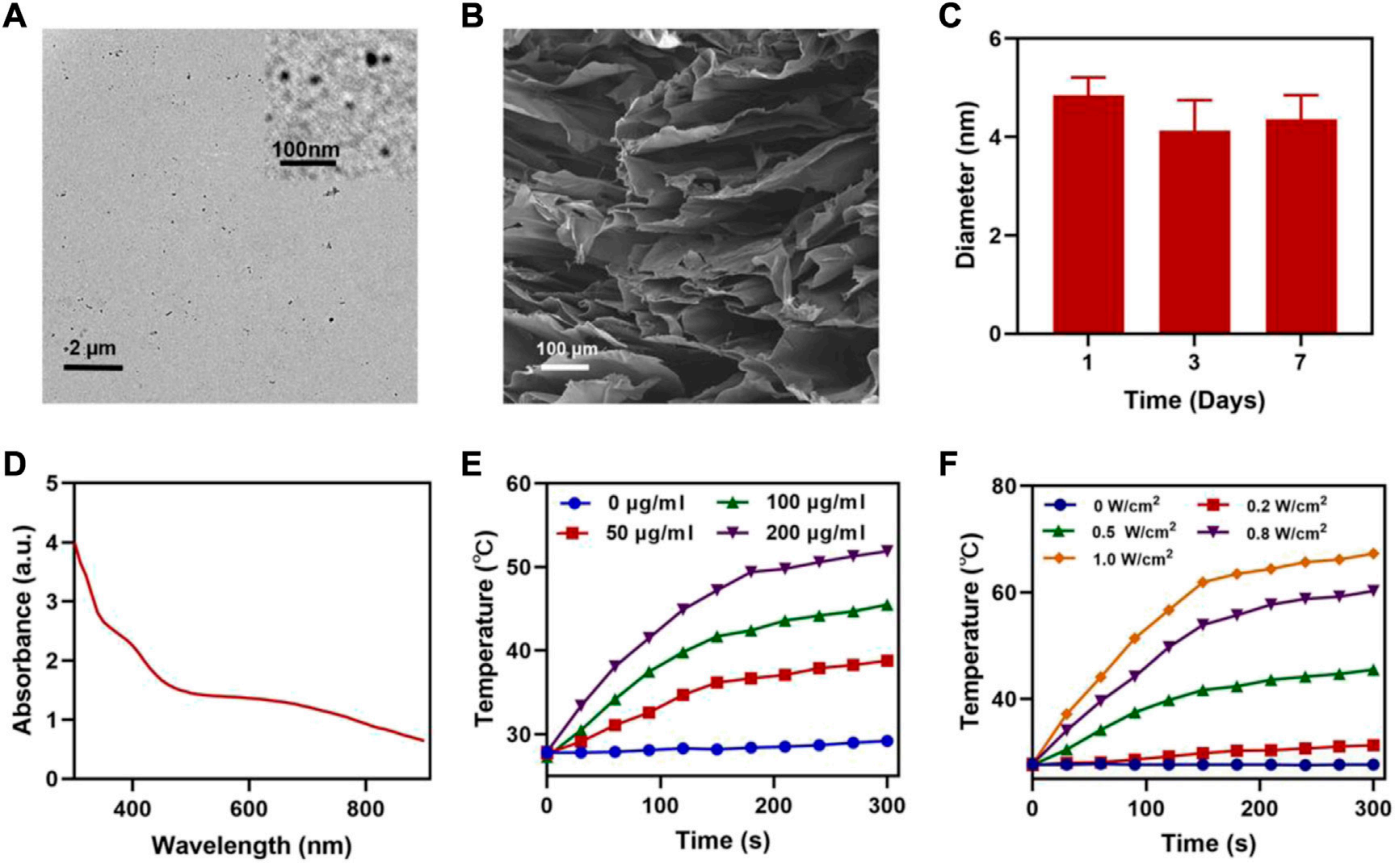
**(A)** TEM image of FeGA NPs (inset FeGA). **(B)** SEM image of FBH. **(C)** Hydrodynamic sizes of FeGA NPs. **(D)** UV-Vis absorption spectrum of FBH. **(E)** Temperature curves of FBH containing FeGA NPs with different concentrations under 808 nm laser at 0.5 W/cm^2^ irradiation. **(F)** Temperature curves of FBH containing 100 μg/ml FeGA NPs under different power densities.

**FIGURE 2:**
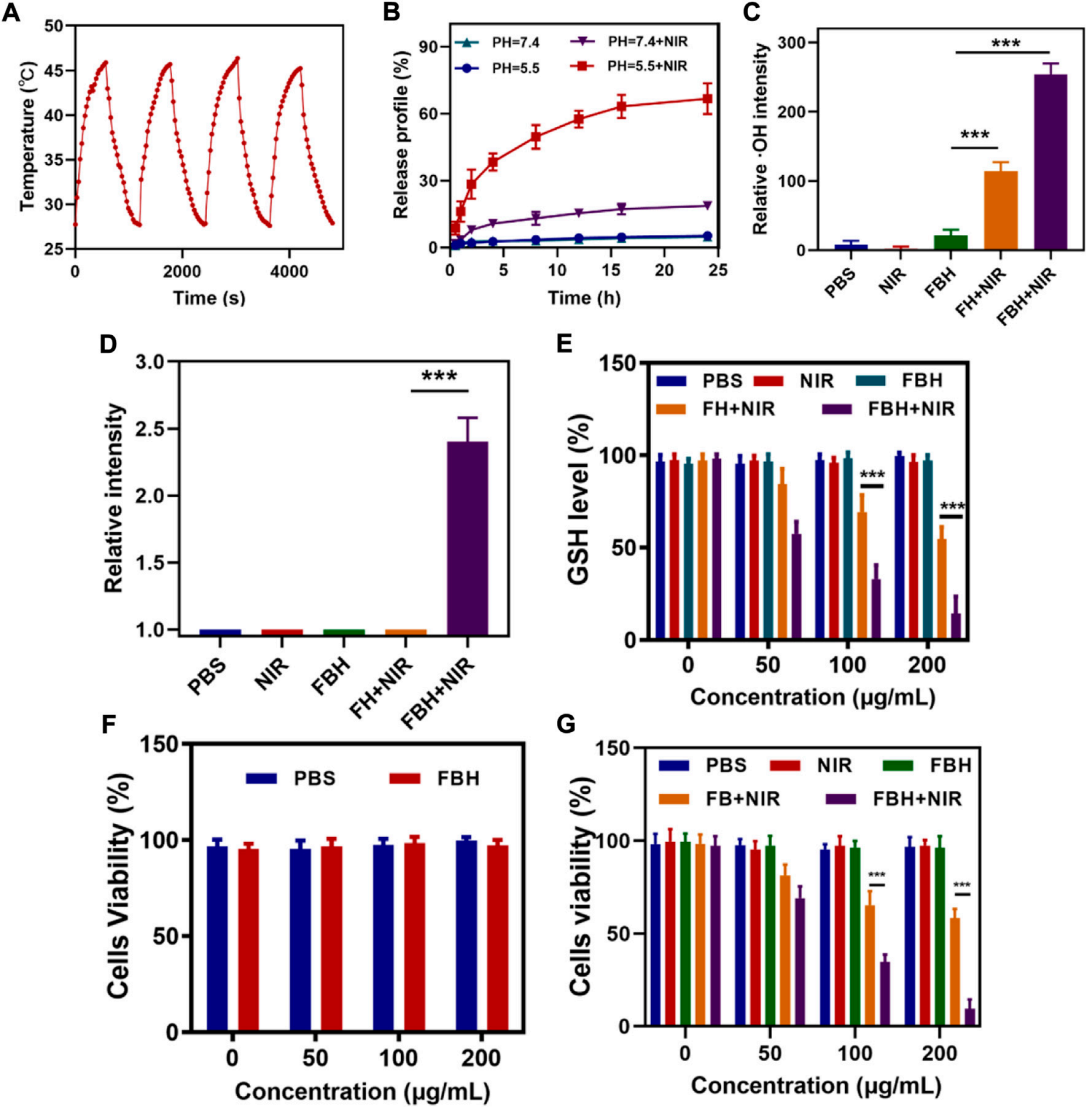
**(A)** Heating curve of FBH under four cycles of heating and cooling processes. **(B)** The release curve of SO_2_ from FBH after different treatments. **(C)** Relative OH intensity in cells after treated by different experimental groups (n = 3). **(D)** Relative SO_2_ intensity in cells after treated by different experimental groups (n = 3). **(E)** The effect of different formulations on the intracellular GSH levels was determined by employing a GSH assay kit (n = 3). **(F)** Cell viabilities of control group (PBS) and FBH system without NIR irradiation (n = 3). **(G)**Various treatments’ *in vitro* cytotoxicity towards 4T1 cells (n = 3). *p < 0.05, **p < 0.01, ***p < 0.005; Student’s t-test.

We prepared FeGA NPs according to the studies in the literature [(Xin Li et al., 2021)] and showed its transmission electron microscope (TEM) image (Figure 1A), then we synthesized the FBH system by encapsulating the prepared FeGA NPs and BTS in 2% agarose hydrogel, and it was imaged by scanning electron microscopy (SEM) (Figure 1B). We then determined the average hydrated particle size of FeGA NPs by dynamic light scattering (DLS) method (Figure 1C), and the results were in good agreement with TEM. The UV-Vis absorption spectra observed that FBH has broad absorption in the NIR (Figure 1D), which indicates that the metal-organic coordination polymer formed by the coordination of GA with iron ions may have better photothermal properties. The photothermal conversion performance was investigated by preparing FBHs containing FeGA NPs at different concentrations (0, 50, 100, 200 μg/ml) and exposing them to a 808 nm laser at 0.5 W/cm2 for 5 min. The resulting concentration- and time-dependent temperature rise curves are shown in Figure 1E. For example, the temperature of FBH containing 200 μg/ml FeGA NPs increased from 27.9 to 51.9 C after laser irradiation. In addition, the laser power dependent photothermal effect (0, 0.2, 0.5, 0.8, and 1.0 W/cm2) was also demonstrated (Figure 1F). This indicates that the temperature increases of the FBH containing FeGA NPs can be tuned from 27.7°C (0 W/cm2) to 67.3°C (1.0 W/cm2) by controlling the power density of the 808 nm laser, demonstrating the photothermal conversion dependence of performance on laser power density. Notably, the photothermal performance of FBH did not regress significantly during four laser on/off cycles, indicating that the as-prepared FBH has high photothermal stability for photothermal therapy (Figure 2A). These results indicate that FeGA nanoparticles have good photothermal stability. According to the photothermal conversion efficiency formula, the photothermal conversion efficiency of FeGA is 45.7%, which is higher than that of Au nanorods (21%) [(Xiong et al., 2021)]. Therefore, FeGA nanoparticles are an excellent photothermal conversion reagent. In our system, the hydrogel-encapsulated SO2 pre-drug BTS is a crystalline white solid with excellent water solubility. Studies have found that BTS can selectively generate SO2 in the acidic environment of tumors [(Yuan et al., 2021)]. Therefore, we further verified the SO2 release efficiency of the BTS-loaded FBH under light-stimulated acidic environment (Figure 2B). As mentioned before, under laser irradiation, our FBH system will achieve a photothermal response, which causes the agarose hydrogel to melt and release BTS. We found that the SO2 release efficiency of the system was significantly enhanced under acidic conditions. At neutral pH (7.4) the reaction is much slower. But in the absence of light, our FBH is in a solidified state at room temperature, and it is difficult to decompose to release BTS, so no SO2 is produced. This further proves that the FBH system prepared by us can controllably perform gas treatment and subsequent further treatment.

**SCHEME 1:**
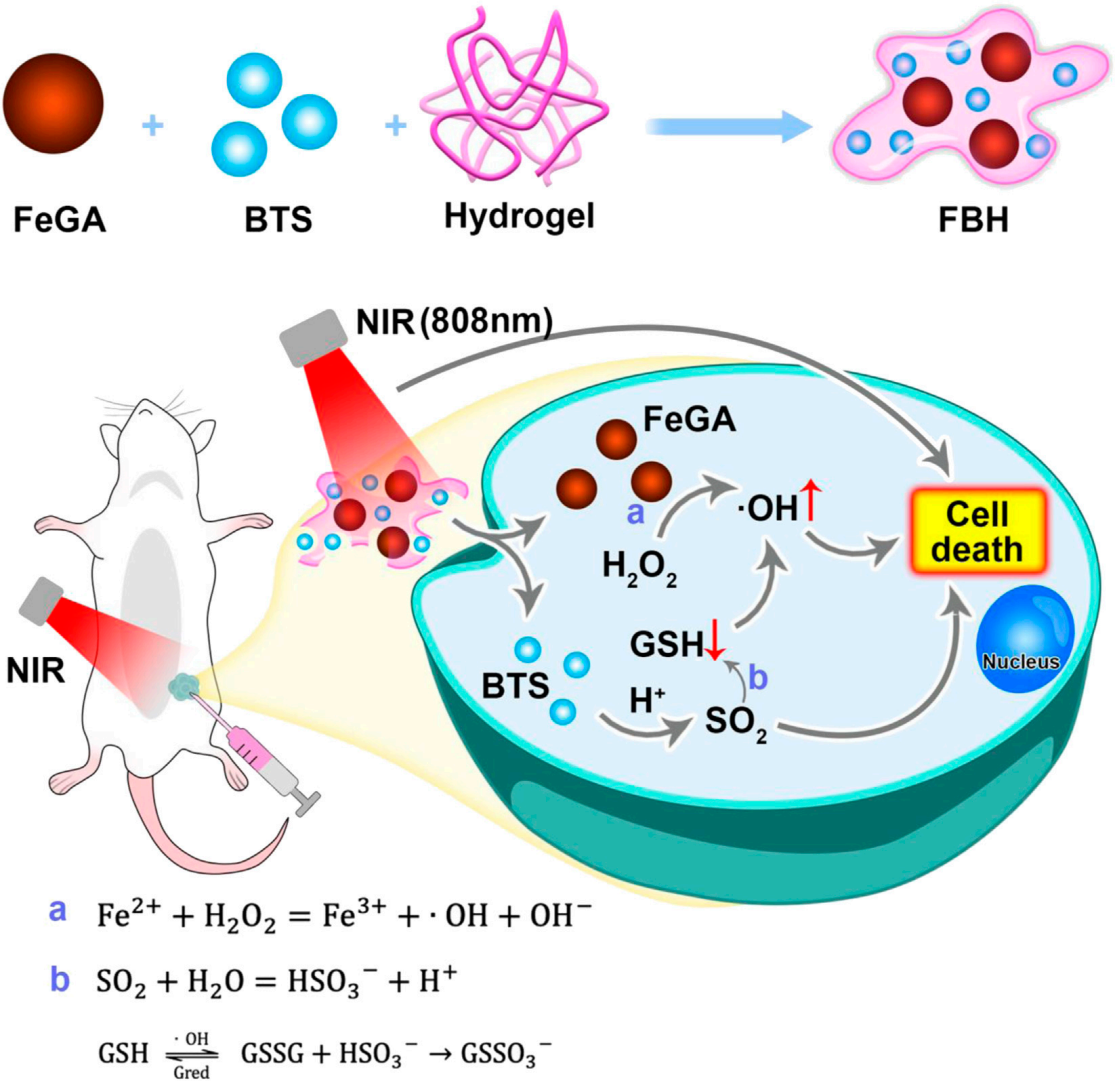
Schematic illustration of hydrogel co-loading SO_2_ prodrug and FeGA NPs for enhancing CDT by photothermal-triggered SO_2_ gas therapy.

As previously shown, FeGA can accelerate the release of Fe^2+^ in an acidic environment and under light, and combine with the excess H_2_O_2_ in the tumor for chemokinetic treatment. To detect FBH-induced ·OH production in cells, we stained 4T1 cells with different treatments using the OH detection probe HPF. As illustrated in Figure 2C, the PBS group, the NIR group and the FBH alone group had almost no OH production, which means that OH was almost absent in normal cells and was not affected by NIR light stimulation, the FBH group alone did not cause cell damage because it was difficult to decompose and release drugs due to the encapsulation of hydrogel. In addition, a hydrogel containing only FeGA NPs (FH) was prepared for exploratory experiments. The FH + NIR group produced OH of moderate intensity, while the FBH + NIR group produced OH of maximum intensity. ROS can cause damage to DNA and cellular protein, leading to tumor cell death. However, in order to maintain cellular redox homeostasis, overexpressed glutathione GSH exists in the tumor microenvironment, and GSH can react with oxidative reactive substances to be oxidized to form oxidized glutathione (GSSG), thus resulting in an inhibition of the anti-tumor effect based on ROS. According to research, the SO_2_ produced by BTS will consume GSSG excessively, and the generated S-sulphoglutathione (GSSO_3_^-^) will be excreted in the form of thiosulfate, thereby disrupting the normal GSH cycle, reducing the GSH content, thereby increasing the level of ROS, changing the redox balance of cells, and producing oxidative stress. Stimulated, eventually triggering apoptosis (Osi et al., 2021). Therefore, we first studied the production of SO_2_ in different treatment groups by cell experiments. As shown in Figure 2D. The results show that only under laser irradiation, our FBH system can melt agarose hydrogel and release BTS to generate SO_2_ due to the photothermal effect. This also proves that our FBH system can be controlled for gas release. We then investigated GSH consumption in different cell treatment groups, as shown in Figure 2E. The results show that FBH and NIR radiation can fully consume GSH. Combined with the conclusion in Figure 2C, it is sufficient to prove that SO_2_ produced by our FBH system can promote the consumption of GSH, which is conducive to the subsequent FeGA-mediated Fenton reaction. In order to evaluate the cytotoxicity of FBH, we employed 4T1 cancer cells to conduct the standard MTT assay (Zuo et al., 2022). As shown in Figure 2F, FBH exhibited little cytotoxicity in the absence of irradiation. Furthermore, we evaluated the antitumor efficacy of FBH through MTT assay (Figure 2G). 4T1 cells were incubated with PBS, NIR (0.5 W/cm^2^, 5 min), FBH, FH + NIR (0.5 W/cm^2^, 5 min) and FBH + NIR (0.5 W/cm^2^, 5 min). From the results, the treatment effects of the control group, the NIR group and the FBH alone group were not satisfactory. In particular, FBH alone showed little cytotoxicity even at the highest doses. This is due to the encapsulation of the hydrogel, and FBH cannot have an effect on the cells. The FBH + NIR group treatment showed more significant cytotoxicity than the FB + NIR group treatment, which may be explained by the photothermal effect opening the SO_2_ release. As anticipated, the FBH + NIR group not only showed the synergistic treatment results of photothermal therapy and gas therapy, but also enhanced FeGA-mediated CDT killing ability of cancer cells by depleting glutathione of cancer cells through SO_2_. This additionally demonstrates that FBH + NIR has good antitumor.

**FIGURE 3:**
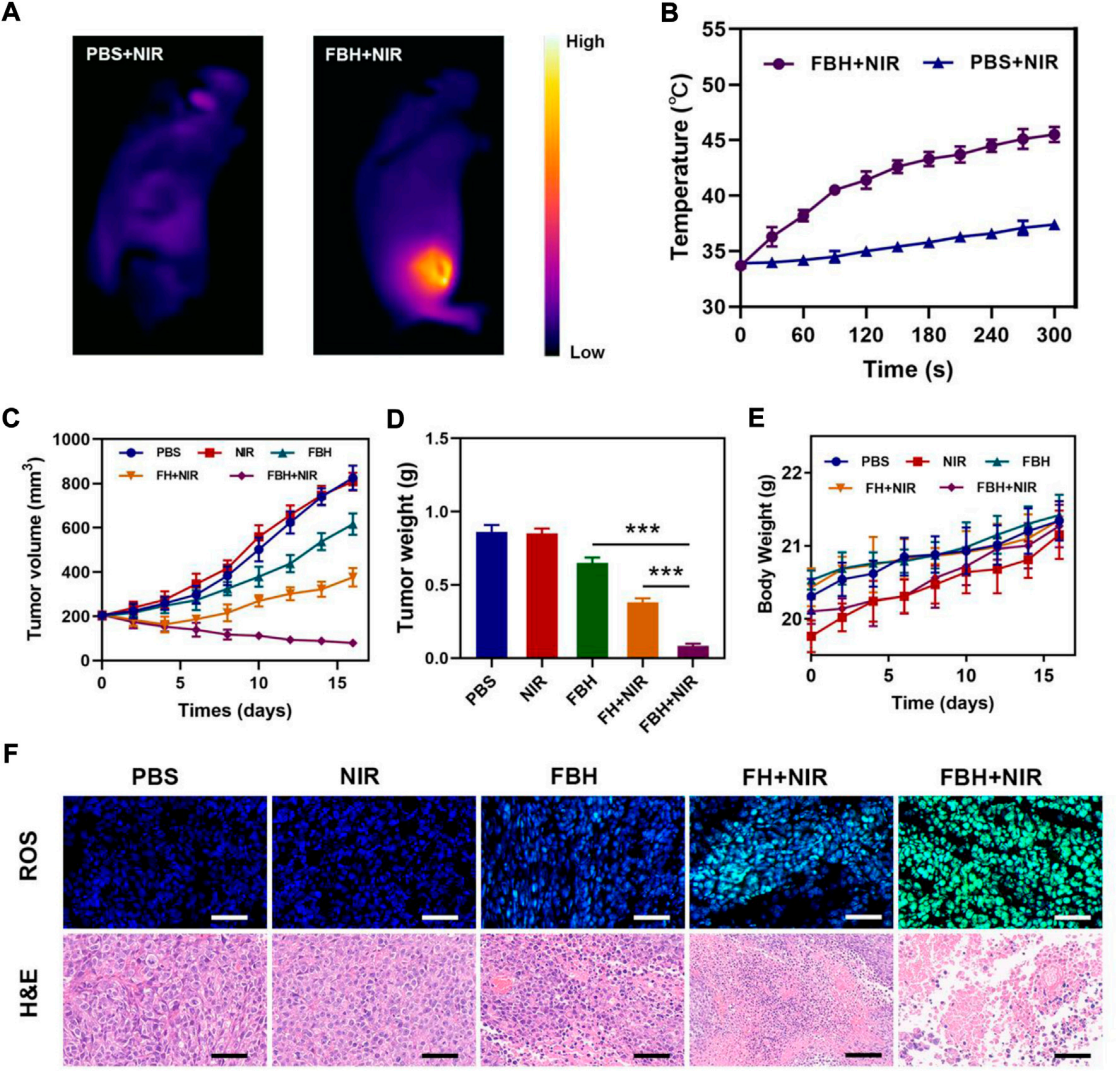
**(A)**Infrared thermal images of tumors in the specified treatment groups. **(B)** After being irradiated with 808 nm laser (0.5 W/cm^2^) for 5 min, the temperature elevates in mice having 4T1 tumor, in the specified treatment groups. **(C)**The treatment groups’ tumor volume alters over time (n = 5). **(D)** Average tumor weight values associated with the indicated treatments (n = 5). **(E)** Changes in body weight in response to the indicated treatments (n = 5). **(F)** HPF and H&E-stained tumor sections from the indicated treatment groups (n = 5). Scale bars: 100 μm *p < 0.05, **p < 0.01, ***p < 0.005; Student’s t-test.

Before studying the antitumor efficacy of FBH *in vivo*, we first studied the photothermal efficacy of the prepared FBH in mice. After intratumoral injection of PBS and FBH, respectively, the mice were exposed to 808 nm (0.5 W/cm^2^) laser light for 5 min, as shown in Figures 3A,B, Photothermal images showed that the tumor temperature in the control group only increased around ΔT = 4°C. In contrast, after light activation, the temperature of the tumor area in mice injected with FBH showed a rapid warming of ΔT = 17°C. This important result suggests that FBH may serve as an excellent therapeutic agent for the synergistic anticancer efficacy of FeGA-mediated CDT/PTT. We then examined the ability of this prepared FBH to induce the elimination of 4T1 mammary tumors in mice as they exhibited positive antitumor activity *in vitro*. We randomly divided unilateral 4T1 tumor-bearing mice with tumor volumes of approximately 200 mm^3^ into five groups: (Zhang et al., 2022a): PBS; (Zhang et al., 2022b); NIR; (Tang et al., 2022); FBH; (Zhang et al., 2022); FH + NIR; (Liu et al., 2021); FBH + NIR. As shown in Figures 3C,D, compared with the PBS, NIR group, the FBH alone group had a slight inhibitory effect on the tumor. Notably, this does not appear to be fully responsive to *in vitro* MTT results. This is because the encapsulated agarose can be slowly decomposed in the body, and the mechanism may be related to the action of some biological enzymes. While the FB + NIR group significantly reduced tumor growth, the FBH + NIR group exhibited higher tumor inhibition rates than FBH and FB + NIR alone. This can be attributed to the synergistic anticancer effects of FeGA-mediated CDT and SO_2_-mediated gas therapy released from FBH in response to light as described above, as well as enhanced CDT killing of cancer cells by disrupting redox homeostasis of the tumor microenvironment through GSH depletion by SO_2_. Furthermore, the high stability of FBH avoids premature drug release in the tumor site. Similarly, none of the groups demonstrated any weight loss during the medication (Figure 3E), indicating that FBH is safe *in vivo*. These findings imply that by photothermally activating the gas treatment, our synthetic FBHs can improve CDT effectiveness against tumor cells. To further explore the antitumor mechanism of FBH, we used the HPF kit to verify the *in vivo* OH production after different treatments (Figure 3F). Green fluorescence was hardly seen in the PBS and NIR groups, and the degradation rate of FBH alone was slow under the action of biological enzymes *in vivo*, so the production of OH was not high. While FH + NIR can also observe obvious fluorescence, but the production of OH is limited because GSH can consume OH. The amount of ROS in the FBH + NIR group was significantly higher than that in the FH + NIR group, showing that SO_2_ can increase the level of intracellular OH. Using H&E staining, the histology of tumor tissue was investigated. The findings demonstrated dense tumor tissue in the PBS and NIR groups, and necrotic tumor cells in the FBH group alone. The FH + NIR group would obviously induce tumor cell necrosis, and its nuclei would shrink or even disappear, while the FBH + NIR group treated tumors. The area of tissue necrosis was larger than that of FBH alone or FH + NIR group, indicating that the killing effect of FBH + NIR on tumor was higher than that of FBH or FH + NIR group. There were hardly any apoptotic cells in the PBS and NIR groups. Taken together, these results indicated that our prepared FBH system could effectively inhibit tumor cell proliferation and promote apoptosis.

**FIGURE 4:**
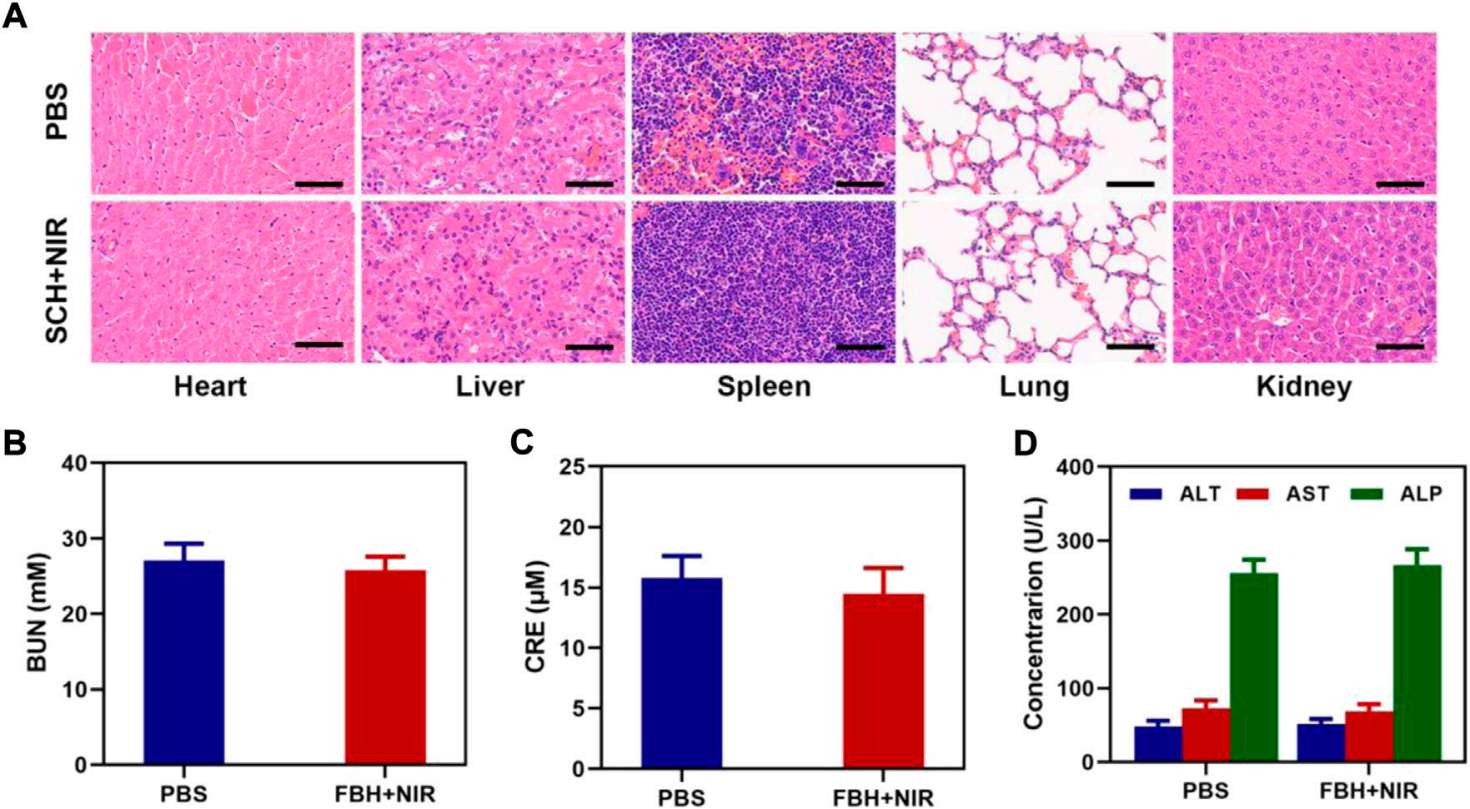
**(A)** Results of H&E-stained images for the mice’s major organs, including the heart, lung, liver, kidneys, and spleen, after exposure to various therapies 16 days after the injection. Scale bars: 100 µm. **(B)** Kidney function markers: BUN, **(C)** CRE, and **(D)** liver function indicators: ALT, AST, and ALP following different treatments.

Additionally, mice in good health were used to test FBH’s long-term biocompatibility. Each group’s mice had their major organs removed and subjected to H&E examination. As seen in Figure 4A, the lung, liver, heart, spleen, and kidney sections of these animals displayed normal histological morphology and no evident pathological alterations, demonstrating that FBH has a low level of systemic toxicity *in vivo*. Blood from FBH-treated mice (*n* = 5) was obtained 16 days after treatment, and hematological and blood biochemical analyses were performed to assess blood cell and major organ function in mice during treatment (Figures 4B–D). Urea (BUN) and creatinine (CRE) are biomarkers of renal function, and alanine aminotransferase (ALT), aspartate aminotransferase (AST), and alkaline phosphatase (ALP) are biomarkers of liver function. These biomarkers did not differ significantly from those of the control group, indicating that the substance did not have any obvious hepatotoxicity or nephrotoxicity. All of the aforementioned findings imply that FBH is a secure medication delivery method with minimal systemic toxicity.

## Conclusion

In summary, we proposed a hydrogel co-loading SO_2_ prodrug and FeGA NPs for enhancing CDT by photothermal-triggered SO_2_ gas therapy system. With the excellent photothermal conversion ability of FeGA NPs and the acidic conditions of the tumor site, BTS can precisely control the release of SO_2_ gas under NIR irradiation. Under NIR irradiation, FeGA could accelerate the release of Fe^2+^ in the acidic tumor microenvironment and react with the excess H_2_O_2_ in the tumor for chemokinetic treatment. At the same time, SO_2_ can deplete GSH in the tumor, which can further enhance the therapeutic impact of Fenton reaction on tumor killing. This FBH system provides a new approach for CDT and SO_2_ gas synergistic therapy and shows excellent anticancer effects *in vivo* and *in vitro*.

## Data Availability

The raw data supporting the conclusions of this article will be made available by the authors, without undue reservation.

## References

[B1] Chang M. Wang M. Wang M. Shu M. Ding B. Li C. (2019). A multifunctional cascade bioreactor based on hollow-structured Cu2 MoS4 for synergetic cancer chemo-dynamic therapy/starvation therapy/phototherapy/immunotherapy with remarkably enhanced efficacy. Adv. Mat. 31 (51), e1905271. 10.1002/adma.201905271 31680346

[B2] Chen X. Chen Y. Wang C. Jiang Y. Chu X. Wu F. (2021). NIR-triggered intracellular H+ transients for lamellipodia-collapsed antimetastasis and enhanced chemodynamic therapy. Angew. Chem. Int. Ed. 60, 21905–21910. 10.1002/anie.202107588 34322970

[B3] Cun J. E. Pan Y. Zhang Z. Lu Y. Li J. Pan Q. (2022). Photo-enhanced upcycling H2O2 into hydroxyl radicals by IR780-embedded Fe3O4@MIL-100 for intense nanocatalytic tumor therapy. Biomaterials 287, 121687. 10.1016/j.biomaterials.2022.121687 35872555

[B4] Deng H. Zhang J. Yang Y. Yang J. Wei Y. Ma S. (2022). Chemodynamic and photothermal combination therapy based on dual-modified metal-organic framework for inducing tumor ferroptosis/pyroptosis. ACS Appl. Mat. Interfaces 14, 24089–24101. 10.1021/acsami.2c00574 35588091

[B5] Fenton H. J. H. (1894). LXXIII.—oxidation of tartaric acid in presence of iron. J. Chem. Soc. Trans. 65 (0), 899–910. 10.1039/CT8946500899

[B6] Fu J. Li T. Yang Y. Jiang L. Wang W. Fu L. (2021). Activatable nanomedicine for overcoming hypoxia-induced resistance to chemotherapy and inhibiting tumor growth by inducing collaborative apoptosis and ferroptosis in solid tumors. Biomaterials 268, 120537. 10.1016/j.biomaterials.2020.120537 33260096

[B7] Guo Q. L. Dai X. L. Yin M. Y. Cheng H. W. Qian H. S. Wang H. (2022). Nanosensitizers for sonodynamic therapy for glioblastoma multiforme: Current progress and future perspectives. Mil. Med. Res. 9 (1), 26. 10.1186/s40779-022-00386-z 35676737 PMC9178901

[B8] Hu H. Yang W. Liang Z. Zhou Z. Song Q. Liu W. (2021). Amplification of oxidative stress with lycorine and gold-based nanocomposites for synergistic cascade cancer therapy. J. Nanobiotechnology 19 (1), 221. 10.1186/s12951-021-00933-1 34315494 PMC8314456

[B9] Li X. Hetjens L. Wolter N. Li H. Shi X. Pich A. (2022c). Charge-reversible and biodegradable chitosan-based microgels for lysozyme-triggered release of vancomycin. J. Adv. Res. [in press]. 10.1016/j.jare.2022.02.014 PMC9811367 36585117

[B10] Li X. Kong L. Hu W. Zhang C. Pich A. Shi X. (2022b). Safe and efficient 2D molybdenum disulfide platform for cooperative imaging-guided photothermal-selective chemotherapy: A preclinical study. J. Adv. Res. 37, 255–266. 10.1016/j.jare.2021.08.004 35499043 PMC9039738

[B11] Li X. Li H. Zhang C. Pich A. Xing L. Shi X. (2021a). Intelligent nanogels with self-adaptive responsiveness for improved tumor drug delivery and augmented chemotherapy. Bioact. Mat. 6 (10), 3473–3484. 10.1016/j.bioactmat.2021.03.021 PMC8024537 33869898

[B12] Li X. Lu S. Xiong Z. Hu Y. Ma D. Lou W. (2019). Light-addressable nanoclusters of ultrasmall iron oxide nanoparticles for enhanced and dynamic magnetic resonance imaging of arthritis. Adv. Sci. 6 (19), 1901800. 10.1002/advs.201901800 PMC6774037 31592427

[B13] Li X. Lu Y. Hu Y. (2022a). A wireless and battery-free DNA hydrogel biosensor for wound infection monitoring. Matter 5 (8), 2473–2475. 10.1016/j.matt.2022.06.021

[B14] Li X. Ouyang Z. Li H. Hu C. Saha P. Xing L. (2021b). Dendrimer-decorated nanogels: Efficient nanocarriers for biodistribution *in vivo* and chemotherapy of ovarian carcinoma. Bioact. Mat. 6 (10), 3244–3253. 10.1016/j.bioactmat.2021.02.031 PMC7970313 33778202

[B15] Li X. Xing L. Hu Y. Xiong Z. Wang R. Xu X. (2017b). An RGD-modified hollow silica@Au core/shell nanoplatform for tumor combination therapy. Acta Biomater. 62, 273–283. 10.1016/j.actbio.2017.08.024 28823719

[B16] Li X. Xing L. Zheng K. Wei P. Du L. Shen M. (2017a). Formation of gold nanostar-coated hollow mesoporous silica for tumor multimodality imaging and photothermal therapy. ACS Appl. Mat. Interfaces 9 (7), 5817–5827. 10.1021/acsami.6b15185 28118704

[B17] Liang L. Wen L. Weng Y. Song J. Li H. Zhang Y. (2021). Homologous-targeted and tumor microenvironment-activated hydroxyl radical nanogenerator for enhanced chemoimmunotherapy of non-small cell lung cancer. Chem. Eng. J. 425, 131451. 10.1016/j.cej.2021.131451

[B18] Lin L. Wang S. Deng H. Yang W. Rao L. Tian R. (2020). Endogenous labile iron pool-mediated free radical generation for cancer chemodynamic therapy. J. Am. Chem. Soc. 142 (36), 15320–15330. 10.1021/jacs.0c05604 32820914

[B19] Lin X. Li L. Li S. Li Q. Xie D. Zhou M. (2021). Targeting the opening of mitochondrial permeability transition pores potentiates nanoparticle drug delivery and mitigates cancer metastasis. Adv. Sci. 8 (4), 2002834. 10.1002/advs.202002834 PMC7887600 33643797

[B20] Liu Y. Zhai S. Jiang X. Liu Y. Wang K. Wang C. (2021). Intracellular mutual promotion of redox homeostasis regulation and iron metabolism disruption for enduring chemodynamic therapy. Adv. Funct. Mat. 31 (17), 2010390. 10.1002/adfm.202010390

[B21] Liu Y. Zhen W. Jin L. Zhang S. Sun G. Zhang T. (2018). All-in-One theranostic nanoagent with enhanced reactive oxygen species generation and modulating tumor microenvironment ability for effective tumor eradication. ACS Nano 12 (5), 4886–4893. 10.1021/acsnano.8b01893 29727164

[B22] Lu Q. Lu T. Xu M. Yang L. Song Y. Li N. (2020). SO2 prodrug doped nanorattles with extra-high drug payload for "collusion inside and outside" photothermal/pH triggered - gas therapy. Biomaterials 257, 120236. 10.1016/j.biomaterials.2020.120236 32738655

[B23] Ma B. Wang S. Liu F. Zhang S. Duan J. Li Z. (2018). Self-assembled copper-amino acid nanoparticles for *in situ* glutathione "AND" H2O2 sequentially triggered chemodynamic therapy. J. Am. Chem. Soc. 141, 849–857. 10.1021/jacs.8b08714 30541274

[B24] Osi A. R. Zhang H. Chen J. Zhou Y. Wang R. Fu J. (2021). Three-dimensional-printable thermo/photo-cross-linked methacrylated chitosan-gelatin hydrogel composites for tissue engineering. ACS Appl. Mat. Interfaces 13 (19), 22902–22913. 10.1021/acsami.1c01321 33960765

[B25] Shen W. Liu W. Yang H. Zhang P. Xiao C. Chen X. (2018). A glutathione-responsive sulfur dioxide polymer prodrug as a nanocarrier for combating drug-resistance in cancer chemotherapy. Biomaterials 178, 706–719. 10.1016/j.biomaterials.2018.02.011 29433753

[B26] Tang Y. Varyambath A. Ding Y. Chen B. Huang X. Zhang Y. (2022). Porous organic polymers for drug delivery: Hierarchical pore structures, variable morphologies, and biological properties. Biomater. Sci. [in press]. 10.1039/d2bm00719c 35861101

[B27] Wang J. Sui L. Huang J. Miao L. Nie Y. Wang K. (2021). MoS2-based nanocomposites for cancer diagnosis and therapy. Bioact. Mat. 6 (11), 4209–4242. 10.1016/j.bioactmat.2021.04.021 PMC8102209 33997503

[B28] Wang X. Zhong X. Bai L. Xu J. Gong F. Dong Z. (2020). Ultrafine titanium monoxide (TiO_1+x_) nanorods for enhanced sonodynamic therapy. J. Am. Chem. Soc. 142 (14), 6527–6537. 10.1021/jacs.9b10228 32191455

[B29] Xin Li H. S. Li H. Hu C. Luo Y. Shi X. Pich A. (2021). Multi-responsive biodegradable cationic nanogels for highly efficient treatment of tumors. Adv. Funct. Mat. 31, 2100227. 10.1002/adfm.202100227

[B30] Xiong Z. Achavananthadith S. Lian S. Madden L. E. Ong Z. X. Chua W. (2021). A wireless and battery-free wound infection sensor based on DNA hydrogel. Sci. Adv. 7 (47), eabj1617. 10.1126/sciadv.abj1617 34797719 PMC8604401

[B31] Xu L. Xu R. Saw P. E. Wu J. Cheng S. X. Xu X. (2021). Nanoparticle-mediated inhibition of mitochondrial glutaminolysis to amplify oxidative stress for combination cancer therapy. Nano Lett. 21 (18), 7569–7578. 10.1021/acs.nanolett.1c02073 34472343

[B32] Yuan H. Han Z. Chen Y. Qi F. Fang H. Guo Z. (2021). Ferroptosis photoinduced by new cyclometalated iridium(III) complexes and its synergism with apoptosis in tumor cell inhibition. Angew. Chem. Int. Ed. 60 (15), 8174–8181. 10.1002/anie.202014959 33656228

[B33] Zhang X. Ong'achwa Machuki J. Pan W. Cai W. Xi Z. Shen F. (2020). Carbon nitride hollow theranostic nanoregulators executing laser-activatable water splitting for enhanced ultrasound/fluorescence imaging and cooperative phototherapy. ACS Nano 14 (4), 4045–4060. 10.1021/acsnano.9b08737 32255341

[B34] Zhang Y. Kim I. Lu Y. Xu Y. Yu D. G. Song W. (2022). Intelligent poly(l-histidine)-based nanovehicles for controlled drug delivery. J. Control. Release 349, 963–982. 10.1016/j.jconrel.2022.08.005 35944751

[B35] Zhang Y. Li S. Xu Y. Shi X. Zhang M. Huang Y. (2022b). Engineering of hollow polymeric nanosphere-supported imidazolium-based ionic liquids with enhanced antimicrobial activities. Nano Res. 15 (6), 5556–5568. 10.1007/s12274-022-4160-6

[B36] Zhang Y. Song W. Lu Y. Xu Y. Wang C. Yu D. G. (2022a). Recent advances in poly(α-L-glutamic acid)-based nanomaterials for drug delivery. Biomolecules 12 (5), 636. 10.3390/biom12050636 35625562 PMC9138577

[B37] Zhao T. Wu W. Sui L. Huang Q. Nan Y. Liu J. (2022). Reactive oxygen species-based nanomaterials for the treatment of myocardial ischemia reperfusion injuries. Bioact. Mat. 7, 47–72. 10.1016/j.bioactmat.2021.06.006 PMC8377441 34466716

[B38] Zhou Q. M. Lu Y. F. Zhou J. P. Yang X. Y. Wang X. J. Yu J. N. (2021). Self-amplification of oxidative stress with tumour microenvironment-activatable iron-doped nanoplatform for targeting hepatocellular carcinoma synergistic cascade therapy and diagnosis. J. Nanobiotechnology 19 (1), 361. 10.1186/s12951-021-01102-0 34749740 PMC8576982

[B39] Zhu D. Chen H. Huang C. Li G. Wang X. Jiang W. (2022a). H_2_O_2_ self-producing single-atom nanozyme hydrogels as light-controlled oxidative stress amplifier for enhanced synergistic therapy by transforming “cold” tumors. Adv. Funct. Mat. 32, 2110268. 10.1002/adfm.202110268

[B40] Zhu D. Duo Y. Meng S. Zhao Y. Xia L. Zheng Z. (2020). Tumor-exocytosed exosome/aggregation-induced emission luminogen hybrid nanovesicles facilitate efficient tumor penetration and photodynamic therapy. Angew. Chem. Int. Ed. 59, 13836–13843. 10.1002/anie.202003672 32367646

[B41] Zhu D. Liu Z. Li Y. Huang Q. Xia L. Li K. (2021b). Delivery of manganese carbonyl to the tumor microenvironment using Tumor-Derived exosomes for cancer gas therapy and low dose radiotherapy. Biomaterials 274, 120894. 10.1016/j.biomaterials.2021.120894 34029916

[B42] Zhu D. Zhang J. Luo G. Duo Y. Tang B. Z. (2021a). Bright bacterium for hypoxia-tolerant photodynamic therapy against orthotopic colon tumors by an interventional method. Adv. Sci. (Weinh). 8, 2004769. 10.1002/advs.202004769 PMC8336512 34145986

[B43] Zhu D. Zhang T. Li Y. Huang C. Suo M. Xia L. (2022b). Tumor-derived exosomes co-delivering aggregation-induced emission luminogens and proton pump inhibitors for tumor glutamine starvation therapy and enhanced type-I photodynamic therapy. Biomaterials 283, 121462. 10.1016/j.biomaterials.2022.121462 35272223

[B44] Zhu Y. Zhao T. Liu M. Wang S. Liu S. Yang Y. (2022c). Rheumatoid arthritis microenvironment insights into treatment effect of nanomaterials. Nano Today 42, 101358. 10.1016/j.nantod.2021.101358

[B45] Zuo H. Qiang J. Wang Y. Wang R. Wang G. Chai L. (2022). Design of red blood cell membrane-cloaked dihydroartemisinin nanoparticles with enhanced antimalarial efficacy. Int. J. Pharm. X. 618, 121665. 10.1016/j.ijpharm.2022.121665 35288223

