# Supplementary material for: Hydrogel co-loading SO2 prodrug and FeGA nanoparticles for enhancing chemodynamic therapy by photothermal-triggered SO2 gas therapy
Source: Front Bioeng Biotechnol. 2022 Sep 29;10:1024089. doi: 10.3389/fbioe.2022.1024089 (PMC9557173; doi:10.3389/fbioe.2022.1024089)
Supplement: Supplementary file 1 [file Table1.DOCX]

Experimental Procedures

**Materials and reagents.**

Iron chloride hexahydrate (FeCl_3_·6H2O) and polyvinylpyrrolidone K30 (PVP) were purchased from Sinopharm Chemical Reagent Co., Ltd. Benzothiazole sulfinate (BTS) was obtained from Duchuang Pharmaceutical Technology Co., Ltd (Shanghai, China). Agarose was purchased from Yare Shanghai. 7-diethylaminocoumarin-3-aldehyde (DEACA) were purchased from Heowns Biochem Technologies Co. Ltd (Tianjin, China). Gallic acid was acquired from Macklin. 3’-(4-hydroxyphenyl) fluorescein (HPF) were purchased from Sigma-Aldrich. The reduced GSH assay kit was purchased from Nanjing Jiancheng Bioengineering Institute. MTT Cell Proliferation Assay Kit was obtained from Yeasen Biotech Co., Ltd (China). The other reagents used in this work were purchased from Sinopharm Chemical Reagent (China) and Aladdin-Reagent (China).

**Cell culture**

4T1 mouse breast cancer cell line was obtained from the Cell Bank of the Chinese Academy of Sciences and incubated in RPMI-1640 medium supplemented with 10% FBS in a humidified atmosphere at 37℃.

**Preparation and characterization of GA(III)-Fe Nanoparticles (FeGA)[**[**1**](#_ENREF_1)**]**

Fresh GA–Fe was prepared according to the literature.([1](#_ENREF_1)) FeCl3·6H2O was added into the gallate solution to keep a 1:1 stoichiometry of Fe^3+^ and GA^4−^ for reaction with magnetic stirring and nitrogen supplementation for 1 h, after which the suspension was centrifuged and the precipitate was washed, collected, and stored for further use. The morphology structures of FeGA were observed by the TEM (JEOL-2100). Dynamic light scattering (DLS) was conducted on a Zeta sizer Nano series (Nano ZS90, Malvern Instrument Ltd.).

**Preparation and characterization of FBH**

The general protocol for the hydrogel preparation is as follows. The prepared FeGA (100µg in 1mL PBS) and BTS (100µg in 1mL PBS) were mixed into 2% agarose solution to form FBH. Scanning electron microscopy (SEM) images were captured on a Hitachi FE-SEM S4800 instrument with an acceleration voltage of 3 kV. UV-vis spectra of samples were recorded by the UV-vis spectrophotometry Lambda 35 (Perkin-Elmer).

**Photothermal Conversion Ability**

An 808 nm NIR laser (Changchun New Industries Tech.Co., Ltd., Changchun, China) with irradiation powers was used to stimulate the concentrations of FeGA (0, 50, 100, 200 ug/mL) in an aqueous medium. The photothermal images of the FeGA-based suspensions during laser irradiation were recorded every 30 s using an infrared thermal imaging system.

The NIR laser with different irradiation powers (0, 0.2, 0.5, 0.8 and 1.0W/cm^2^) was used to stimulate the FeGA concentration of 100ug/mL in an aqueous medium. The photothermal images of the FeGA-based suspensions during laser irradiation were recorded every 30 s using an infrared thermal imaging system.

Heating curve of FBH for four cycles at a power intensity of 0.5 W/cm^2^ under 808nm laser was measured by the infrared thermal imaging system.

**SO_2_ release study**

For the qualitative investigation of SO_2_ release by FBH, the fluorescence spectrophotometer was utilized to measure the fluorescence intensity according to previous report. BTS-induced SO_2_ generation could be attributed to the cleavage of the C-S bond of BTS in low pH. The generated SO_2_ could be detected by 7-diethylaminocoumarin-3-aldehyde (DEACA), which could react with SO_2_ to show enhanced fluorescence signal at 483 nm. DEACA was selected as a fluorescent probe for bisulfite, which would change from non-fluorescent to blue fluorescent selectively owing to the nucleophilic addition reaction to the formyl functional group with bisulfite anion. Fresh 5 μm of DEACA solution was added into the aqueous solutions of 100 μg/mL FBH in aqueous solution (pH 7.4, 5.5) with or without laser irradiation (808 nm, 0.5 W/cm^2^) for 5 min.

***In vitro* ROS generation**

ROS generation was also assessed in vitro on 4T1 cells. The intracellular generation

of ·OH was detected utilizing HPF detection kit. Afterwards, cells were incubated for 5 different groups: (1) PBS; (2) NIR; (3) FBH; (4) FH + NIR; (5) FBH + NIR. The FeGA concentration was 100 μg/mL in group 3，4 and 5. Then, HPF detection kit was added. Then, cells in group 2, 4 and 5 were exposed to 808 nm laser radiation (0.5 W/cm^2^) for 5 min and detected under a fluorescent microscope (IX81, Olympus, Japan). Fluorescence intensity was measured by ImageJ software.

***In vitro* SO_2_ release**

For the qualitative study of SO2 release from cells after treatment in different experimental groups, DEACA was also selected as the fluorescent probe of bisulfite.4T1 cells were incubated with 5 different group: (1) PBS; (2) NIR; (3) FBH; (4) FH + NIR; (5) FBH + NIR. The FBH concentration in groups 3, 4, and 5 was μg/mL. Then, cells in group 2, 4 and 5 were exposed to 808 nm laser radiation (0.5 W/cm2) for 5 min. After 12 hours of incubation, the cells were lysed with cell lysing buffer, and then the supernatant was collected by centrifugation, and 5 μm of fresh DEACA solution was added, and the supernatant was allowed to stand for 5 minutes. The fluorescence intensity was measured using a fluorescence spectrophotometer.

**Detection of Intracellular GSH.**

The commercially available GSH assay kit was used to detect the depletion of GSH. 4T1 cells were incubated with 5 different group at different FeGA concentration (0, 50, 100 and 200 μg/mL): (1) PBS; (2) NIR; (3) FBH; (4) FH + NIR; (5) FBH + NIR. Then, cells in group 2, 4 and 5 were exposed to 808 nm laser radiation (0.5 W/cm^2^) for 5 min. After 12 hours of incubation, the GSH content was measured by employing a commercial colorimetric GSH assay kit. The assay was carried out according to the manufacturer’s instructions. The absorbance of 340 nm was measured by a microplate reader.

***In vitro* anti-cancer effect of FBH**

The phototoxicity was measured by MTT assay. 4T1 cells were seeded in 96-well plates at a density of 5 × 10^3^ cells per well and incubated for 24 h. Afterwards, 4T1 cells were incubated with 5 different groups: (1) PBS; (2) Near infrared laser irradiation (808nm laser, 0.5W/cm^2^); (3) FBH; (4) FH+NIR and (5) FBH+NIR. Afterwards, cells were incubated for 6h with different concentrations of FeGA (0, 50, 100 and 200 μg/mL). Then, cells in group 2, 4 and 5 were irradiated with the NIR. At the end of the incubation, 5 mg/mL MTT solution was added, and the plate was incubated for another 4 h. Finally, the absorbance values of the cells were determined by using a microplate reader (Emax Precision, USA) at 570 nm. The background absorbance of the well plate was measured and subtracted. The cytotoxicity was calculated by dividing the optical density (OD) values of treated groups (T) by the OD values of the control (C) (T/C × 100%).

**Animal tumor models**

Female BALB/c nude mice aged 4-5 week were purchased from Vital River Company (Beijing, China). 100 μL of 4T1 cell suspension (1×10^6^ cells per mL) were subcutaneous injected into each mouse to establish the tumor models. The animal experiments were carried out according to the protocol approved by the Ministry of Health in People’s Republic of PR China and were approved by the Administrative Committee on Animal Research of the Wuhan University.

***In vivo* infrared thermography**

To monitor the photothermal effect in vivo, FBH (FeGA: 10 mg/kg, BTS: 10 mg/kg) was injected into tumor-bearing mice, and 0.5 W/cm^2^ was irradiated 1 h after injection for 5min. The control group was PBS injection. An infrared camera (Fotric 225) was used to image mice before and after irradiation. At the same time, the temperature of the tumor was monitored every 30s using an infrared camera.

***In vivo* antitumor study**

The mice were firstly divided randomly into 5 groups (each group included 5 mice): (1) PBS; (2) NIR; (3) FBH; (4) FH + NIR; (5) FBH + NIR. The FeGA dose was 10 mg/kg in group 3, 4, and 5. Then, cells in group 2, 4 and 5 were exposed to 808 nm laser radiation (0.5 W/cm^2^) for 5 min. The injection method is intratumoral injection. NIR was conducted 1h after the injection. Mice body weight and tumor volume in all groups were monitored every 2 days. A caliper was employed to measure the tumor length and tumor width and the tumor volume was calculated according to following formula. Tumor volume = tumor length × tumor width^2^ / 2. After 15 days treatment, mice were sacrificed. Five main organs (heart, liver, spleen, lung and kidney) of all mice were harvested, washed with PBS, and fixed with paraformaldehyde for histology analysis. The blood samples from these mice (≈1 mL) were collected for blood biochemistry analysis. And the tumor tissues were weighed, and fixed in 4% neutral buffered formalin, processed routinely into paraffin, and sectioned at 4 μm. Then the sections were stained with HPF and H&E and finally examined by using optical microscope (BX51, Olympus, Japan) and fluorescence microscope (IX81, Olympus, Japan).

**Statistical analysis**

Data analyses were conducted using the GraphPad Prism 5.0 software. Significance between every two groups was calculated by the student’s t-test. *P < 0.05, **P < 0.01, ***P < 0.005.
